# Liver resection and ablation for squamous cell carcinoma liver metastases

**DOI:** 10.1093/bjsopen/zrab060

**Published:** 2021-08-24

**Authors:** J Engstrand, L F Abreu de Carvalho, D Aghayan, A Balakrishnan, A Belli, B Björnsson, B V M Dasari, O Detry, M Di Martino, B Edwin, J Erdmann, R Fristedt, G Fusai, T Gimenez-Maurel, O Hemmingsson, C Hidalgo Salinas, B Isaksson, A Ivanecz, F Izzo, W T Knoefel, P Kron, N Lehwald-Tywuschik, M Lesurtel, J P A Lodge, N Machairas, M V Marino, V Martin, A Paterson, J Rystedt, P Sandström, A Serrablo, A K Siriwardena, H Taflin, T M van Gulik, S Yaqub, İ Özden, J M Ramia, C Sturesson, on behalf of the E-AHPBA Scientific and Research Committee

**Affiliations:** Division of Surgery, Department of Clinical Sciences, Karolinska Institutet at Danderyd Hospital, Stockholm, Sweden; Department of Hepatopancreatobiliary Surgery and Liver Transplantation, Ghent University Hospital, Ghent, Belgium; The Intervention Centre, Oslo University Hospital, Institute of Clinical Medicine, Medical Faculty, University of Oslo, Oslo, Norway; Department of Surgery N1, Yerevan State Medical University after M. Heratsi, Yerevan, Armenia; Department of Surgery, Cambridge University Hospitals NHS Foundation Trust, Cambridge, UK; Department of Abdominal Oncology, HPB Surgical Oncology Unit, National Cancer Institute, Fondazione G. Pascale–IRCCS, Naples, Italy; Department of Surgery in Linköping, Department of Biomedical and Clinical Sciences, Linköping University, Linköping, Sweden; Department of Hepatobiliary and Pancreatic Surgery, Queen Elizabeth Hospital, Birmingham, UK; Department of Abdominal Surgery and Transplantation, CHU Liège, Liège, Belgium; HPB Unit, Department of General and Digestive Surgery, Hospital Universitario La Princesa, Instituto de Investigación Sanitaria Princesa (IIS-IP), Universidad Autónoma de Madrid (UAM), Madrid, Spain; The Intervention Centre, Oslo University Hospital, Institute of Clinical Medicine, Medical Faculty, University of Oslo, Oslo, Norway; Department of Surgery, Cancer Centre Amsterdam, Amsterdam UMC, University of Amsterdam, the Netherlands; Department of Surgery, Skåne University Hospital, Lund University, Lund, Sweden; Department of HPB and Liver Transplant Surgery, Royal Free Hospital, NHS Foundation Trust, London, UK; Department of Surgery, Miguel Servet University Hospital, Zaragoza, Spain; Department of Surgical and Perioperative Sciences, Umeå University, Umeå, Sweden; Department of HPB and Liver Transplant Surgery, Royal Free Hospital, NHS Foundation Trust, London, UK; Department of Surgical Sciences, Uppsala University, Uppsala, Sweden; Department of Abdominal and General Surgery, University Medical Centre Maribor, Maribor, Slovenia; Department of Abdominal Oncology, HPB Surgical Oncology Unit, National Cancer Institute, Fondazione G. Pascale–IRCCS, Naples, Italy; Department of Surgery (A), Heinrich-Heine-University and University Hospital Düsseldorf, Düsseldorf, Germany; Department of Hepatobiliary Surgery, Leeds Teaching Hospitals NHS Trust, Leeds, UK; Department of Surgery (A), Heinrich-Heine-University and University Hospital Düsseldorf, Düsseldorf, Germany; Department of Digestive Surgery and Liver Transplantation, Croix-Rousse University Hospital, Hospices Civils de Lyon, University of Lyon I, Lyon, France; Department of Hepatobiliary Surgery, Leeds Teaching Hospitals NHS Trust, Leeds, UK; 3rd Department of Surgery, National and Kapodistrian University of Athens, Attikon University Hospital, Athens, Greece; General Surgery Department, Azienda Ospedaliera Ospedali Riuniti Villa Sofia-Cervello, Palermo (PA), Abano, Italy; General Surgery Department, Policlinico Abano Terme, Abano, Italy; Department of Digestive Surgery and Liver Transplantation, Croix-Rousse University Hospital, Hospices Civils de Lyon, University of Lyon I, Lyon, France; Department of Surgery, Cambridge University Hospitals NHS Foundation Trust, Cambridge, UK; Department of Surgery, Skåne University Hospital, Lund University, Lund, Sweden; Department of Surgery in Linköping, Department of Biomedical and Clinical Sciences, Linköping University, Linköping, Sweden; Department of Surgery, Miguel Servet University Hospital, Zaragoza, Spain; Hepatobiliary Surgery Unit, Manchester Royal Infirmary, Manchester, UK; Department of Surgery, Institute of Clinical Sciences, Sahlgrenska Academy at University of Gothenburg, Sahlgrenska University Hospital, Sweden; Department of Surgery, Cancer Centre Amsterdam, Amsterdam UMC, University of Amsterdam, the Netherlands; Department of Hepato-Pancreato-Biliary Surgery, Oslo University Hospital, Oslo, Norway; Department of General Surgery, Istanbul University School of Medicine, Istanbul, Turkey; Hospital General Universitario de Alicante. ISABIAL Alicante, Spain; Division of Surgery, Department of Clinical Science, Intervention and Technology (CLINTEC), Karolinska Institutet and Karolinska University Hospital, Stockholm, Sweden

## Abstract

**Background:**

Limited evidence exists to guide the management of patients with liver metastases from squamous cell carcinoma (SCC). The aim of this retrospective multicentre cohort study was to describe patterns of disease recurrence after liver resection/ablation for SCC liver metastases and factors associated with recurrence-free survival (RFS) and overall survival (OS).

**Method:**

Members of the European–African Hepato-Pancreato-Biliary Association were invited to include all consecutive patients undergoing liver resection/ablation for SCC liver metastases between 2002 and 2019. Patient, tumour and perioperative characteristics were analysed with regard to RFS and OS.

**Results:**

Among the 102 patients included from 24 European centres, 56 patients had anal cancer, and 46 patients had SCC from other origin. RFS in patients with anal cancer and non-anal cancer was 16 and 9 months, respectively (*P* = 0.134). A positive resection margin significantly influenced RFS for both anal cancer and non-anal cancer liver metastases (hazard ratio 6.82, 95 per cent c.i. 2.40 to 19.35, for the entire cohort). Median survival duration and 5-year OS rate among patients with anal cancer and non-anal cancer were 50 months and 45 per cent and 21 months and 25 per cent, respectively. For the entire cohort, only non-radical resection was associated with worse overall survival (hazard ratio 3.21, 95 per cent c.i. 1.24 to 8.30).

**Conclusion:**

Liver resection/ablation of liver metastases from SCC can result in long-term survival. Survival was superior in treated patients with liver metastases from anal *versus* non-anal cancer. A negative resection margin is paramount for acceptable outcome.

## Teaser

Limited evidence exists to guide the management of patients with liver metastases from squamous cell carcinoma (SCC). The present study shows that hepatic resection/ablation of liver metastases from SCC can result in long-term survival.

## Introduction

There is limited evidence to guide the management of patients with liver metastases from squamous cell carcinoma (SCC). In metastatic SCC, spread to the liver seems only possible through systemic haematogenous dissemination, which explains the historical concern that hepatectomy is less beneficial for these patients. During the past two decades, several publications have reported on liver resection for non-colorectal non-neuroendocrine liver metastases, unanimously concluding that it is a safe treatment option with the potential of long-term survival^1–5^. These studies have in common the limited number of patients with SCC, and since the group of patients with non-colorectal non-neuroendocrine liver metastases constitutes a highly heterogeneous group with regards to primary tumour location, histology, biology, metastatic pathways, treatment strategies and outcome, analysing factors influencing outcome in the subpopulation with SCC liver metastasis is problematic.

The aim of this retrospective multicentre study was to describe patterns of disease recurrence after liver resection/ablation of SCC liver metastases and factors associated with recurrence-free survival (RFS) and overall survival (OS) through collaboration within the European–African Hepato-Pancreato-Biliary Association (E-AHPBA).

## Methods

Members of the E-AHPBA were invited to include retrospectively all consecutive patients submitted to liver resection and/or ablation of histologically proven SCC liver metastases between 2002 and 2019. The study protocol was approved by the regional ethics board in Stockholm, Sweden (Dnr 2019–04681). All participating centres obtained ethical approval according to national/local legislation before inclusion of patients. Participating centres entered all data into a web-based application for collecting data, Research Electronic Data Capture (REDCap, Stockholm, Sweden), containing predefined case report forms designed specifically for this project to collect relevant data.

The following data were recorded for each patient: age, sex, ASA physical status, Eastern Cooperative Oncology Group (ECOG) performance status and Charlson co-morbidity index^6^. Details and treatment strategy of the primary tumour were collected from medical records. The number, location and size of liver metastases were recorded as well as chemotherapy strategy for metastatic disease and perioperative data concerning the liver resection/thermal ablation. Any recurrence, site of recurrence and potential re-treatments were recorded. Synchronous detection of liver metastases was defined as metastases diagnosed within 3 months of primary tumour diagnosis. Radical resection, R0, was defined as a 1-mm tumour-free margin. A positive resection margin was defined as R1. Complications were recorded up to 30 days postoperatively and classified according to Clavien–Dindo classification, where grades IIIb–IV were defined as a major complication^7^. The patients were censored at date of last follow-up. Analyses were performed for the entire group of included patients and separately for patients with anal cancer liver metastases.

### Statistical analysis

Descriptive statistics were used to depict the study cohort. Continuous variables were described as median (non-normally distributed data) with range and differences tested with Wilcoxon rank-sum test. Categorical variables were specified with frequencies (percentage) and differences in proportions were analysed with Pearson’s χ^2^ test or Fisher’s exact test, the latter if sample sizes were small (10 patients or fewer). RFS was measured from date of liver resection/ablation until date of first recurrence, detected on any radiography, or death. Patients with simultaneously diagnosed extrahepatic metastases that were not treated with curative intent were excluded from the analysis of RFS and factors influencing RFS. OS was measured from date of liver resection/ablation until date of death or date of last follow-up. Survival probabilities were illustrated using Kaplan–Meier graphs and the log rank test for testing equality of survival functions between groups. Patient and tumour factors potentially influencing RFS and OS were analysed using Cox proportional hazard regression model and included in the multivariable analysis if *P* < 0.200 in the univariable analysis, and reported as hazard ratio with associated 95 per cent confidence intervals. For the purposes of data analysis in the regression models, values were dichotomized based on median values. Statistical significance was set at a two-sided alpha level of 0.050. All statistical analyses were performed in STATA 15.0 (StataCorp, College Station, Texas, USA).

## Results

Some 102 patients were included from 24 European institutions; 80 of these patients were diagnosed and treated for liver metastases after 2010. There were 56 patients with anal cancer liver metastases and 46 patients with non-anal SCC liver metastases. The baseline patient and tumour characteristics are summarized in *Tables 1* and *2*.

**Table 1:** Clinicopathological characteristics in patients with liver metastases from squamous cell carcinoma

	Anal cancer liver metastases (*n* = 56)	Non-anal cancer liver metastases (*n* = 46)
Patient characteristics		
Age (years)*	59 (29–81)	58 (37–82)
Gender, women	41 (73)	21 (46)
ASA class		
1–2	44 (79)	30 (65)
3	9 (16)	13 (28)
Missing	3 (5)	3 (7)
ECOG performance status		
0–1	45 (80)	27 (59)
2–3	6 (11)	13 (28)
Missing	5 (9)	6 (13)
BMI (kg/m^2^)*	25.9 (18.0–39.7)	23.9 (15.7–32.0)
CCI at diagnosis of liver metastases		
6–8	32 (57)	21 (46)
9–13	18 (32)	24 (52)
Missing	6 (11)	1 (2)
Primary tumour		
Origin		
Head and neck	NA	12 (26)
Lung	NA	8 (18)
Oesophagus	NA	7 (15)
Gallbladder	NA	6 (13)
Cervix	NA	6 (13)
Vagina	NA	2 (4)
Prostate	NA	1 (2)
Unknown/other	NA	4 (9)
Stage of primary tumour		
T1–T2	21 (38)	26 (57)
T3–T4	31 (55)	17 (37)
Missing	4 (7)	3 (6)
Nodal status of primary tumour		
Negative	24 (43)	17 (37)
Positive	28 (50)	23 (50)
Missing	4 (7)	6 (13)
Liver metastases		
Presentation		
Synchronous	16 (29)	19 (41)
Metachronous	40 (71)	27 (59)
Number of liver metastases*	2 (1–10)	1 (1–4)
Size of largest liver metastasis (mm)*	31 (5–170)	34.5 (6–140)
Number of involved segments		
1	16 (29)	18 (39)
2 or more	36 (64)	26 (57)
Missing	4 (7)	2 (4)
Distant metastases at diagnosis of liver metastases, yes		
Lung	7 (13)	9 (20)
Lymph nodes	2	1
Peritoneum	0	3
Bone	0	2
Other	0	2

Values in parentheses are percentages unless indicated otherwise;

*values are median (range). ECOG, Eastern Cooperative Oncology Group; CCI, Charlson co-morbidity index; NA, not applicable.

**Table 2:** Treatment details in patients with liver metastases from squamous cell carcinoma

	Anal cancer liver metastases (*n* = 56)	Non-anal cancer liver metastases (*n* = 46)
Treatment of primary tumour		
Chemotherapy + radiotherapy	46 (82)	9 (20)
Chemotherapy + radiotherapy + surgery	7 (12)	9 (20)
Radiotherapy	2 (4)	2 (4)
Radiotherapy + surgery	1 (2)	4 (9)
Resection only	0	13 (28)
Resection + chemotherapy	0	7 (15)
No treatment of primary tumour	0	2 (4)
Treatment of liver metastases		
Surgery		
Resection only	48 (86)	38 (83)
Resection + thermal ablation	6 (11)	4 (9)
Thermal ablation only	2 (4)	3 (6)
Resection + pancreaticoduodenectomy and hemicolectomy	0	1 (2)
Type of liver resection		
Wedge	19 (35)	16 (37)
Segmentectomy	17 (31)	16 (37)
Hemihepatectomy	12 (22)	8 (19)
Trisectionectomy	3 (6)	1 (2)
Other	3 (6)	2 (5)
Surgical approach		
Open	40 (74)	33 (77)
Laparoscopic	14 (26)	10 (23)
Complications according to Clavien–Dindo		
Minor complications	19 (34)	28 (61)
Major complications	4 (7)	5 (11)
Resection margin of liver metastases		
R0	47 (87)	37 (86)
R1	7 (13)	6 (14)
Systemic chemotherapy		
None	21 (38)	20 (44)
Neoadjuvant	27 (48)	13 (28)
Adjuvant	7 (12)	11 (24)
Unspecified	1 (2)	2 (4)
Systemic chemotherapy regimen		
5-fluorouracil + mitomycin	7 (20)	0
5-fluorouracil + platinum-based	23 (66)	8 (31)
Platinum-based + other	1 (3)	15 (58)
Other	4 (11)	3 (11)
Site of first recurrence after liver resection/ablation		
None	23 (41)	14 (30)
Intrahepatic only	6 (10)	5 (11)
Extrahepatic only	11 (20)	10 (22)
Intra- and extrahepatic	16 (29)	14 (30)
Unknown	0	3 (7)

Values in parentheses are percentages

Chemotherapy was administered to 61 out of 102 patients; details on regimen strategy and other perioperative characteristics for the entire cohort are summarized in *Table 2*. A higher proportion of patients with synchronous liver metastases (74 *versus* 52 per cent, *P* = 0.031) and multiple liver metastases (74 *versus* 50 per cent, *P* = 0.016) were treated with systemic chemotherapy while primary tumour origin (anal *versus* non-anal, *P* = 0.540), extrahepatic metastases (*P* = 0.811) and nodal status of primary (*P* = 0.524) did not influence chemotherapy strategy. Response to chemotherapy was reported for 37 of 61 patients: nine patients had stable disease, 24 had partial response and four patients had tumour progression.

Treatment with curative intent of simultaneously detected extrahepatic metastases was given to 11 of 16 patients. The five patients whose extrahepatic metastases eventually were not treated with curative intent originated from anal cancer (1 patient), cervical cancer (2 patients) and gallbladder cancer (2 patients).

Liver resection only was carried out in 87 patients, 10 patients had a combination of resection and thermal ablation and five patients underwent thermal ablation only. Median operative time was 196 (range 40–580) minutes). Major complications occurred in nine patients within 30 days of surgery (*Table 2*). Median length of hospital stay was 7 (range 0–87) days). Four patients died within 90 days of surgery of which one death was a direct consequence of the surgical interventions (Clavien–Dindo V).

During a median follow-up time of 22 (range 0.5–145) months from liver resection/ablation, recurrent liver metastases in the entire cohort were diagnosed in 42 patients. The metastases were solely intrahepatic in 11 patients and intrahepatic metastasis was associated with extrahepatic metastasis in 30 patients. Of those with any liver recurrence (42 patients), 15 patients had a second hepatectomy. A further 21 patients suffered from extrahepatic recurrence only, including recurrence at primary tumour site, and none of them had surgery for these extrahepatic metastases or re-resection of primary site recurrences. The extrahepatic recurrences (51 patients) were most often first diagnosed in the lung (24 patients), followed by distant lymph node metastases (2 patients), peritoneum (6 patients), bone (5 patients), brain (3 patients) and other (8 patients); extrahepatic recurrences were in a single organ in 31 of 51 patients and were diagnosed at multiple locations in the remaining 20 patients.

In the subgroup of patients with anal cancer liver metastases (56 patients), 22 had intrahepatic recurrence after liver resection, five had simultaneously diagnosed extrahepatic recurrence and a further 11 patients later developed extrahepatic recurrence. Of these 22 patients, 11 had a repeat liver resection of which nine were considered to have curative intent.

**Fig. 1:**
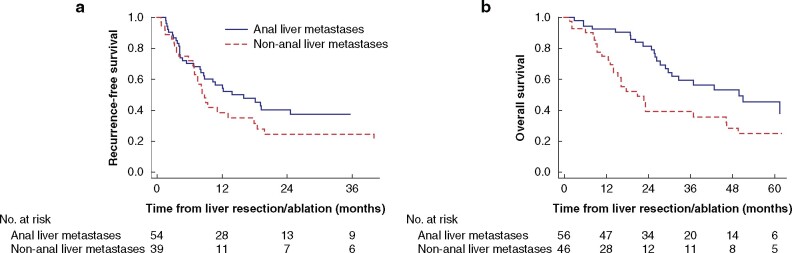
Kaplan–Meier plots showing survival for patients with anal and non-anal squamous cell carcinoma liver metastases undergoing liver resection or ablation **a** Recurrence-free survival. **b** Overall survival. Survival for patients with anal cancer was superior to that of patients with non-anal cancer (*P* = 0.023)

The median RFS after liver resection in the entire cohort (excluding five patients whose simultaneous extrahepatic metastases were not treated curatively and four patients with missing data on recurrence) was 11 (95 per cent c.i. 8 to 19) months, as outlined in *Table 3*. In patients resected for liver-metastatic anal cancer, the median RFS was 16 (95 per cent c.i. 8 to not reached) months and in the non-anal cancer group the median RFS was 9 (95 per cent c.i. 7 to 18) months, log rank test *P* = 0.134 (*Fig. 1a*). The only factor significantly influencing RFS among patients resected for liver-metastatic anal cancer was a positive resection margin (hazard ratio 6.90, 95 per cent c.i. 2.33 to 20.50) (*Table 4*). Factors independently influencing RFS negatively in the entire cohort (93 patients) were an R1 resection (hazard ratio 6.82, 95 per cent c.i. 2.40 to 19.35) and non-anal primary (hazard ratio 2.08, 95 per cent c.i. 1.10 to 3.96).

**Table 3:** Recurrence-free survival and overall survival in liver-metastatic squamous cell carcinoma

	Recurrence-free survival		Overall survival				
	No. of patients	Median RFS (months)	No. of patients	Median survival (months)	1-year OS rate (%)	3-year OS rate (%)	5-year OS rate (%)
**Total cohort**	93	11.1 (8.2–18.5)	102	36.7 (25.7–49.7)	85.0	50.7	36.3
**Anal cancer**	54	15.9 (8.0–NR)	56	49.7 (29.6–NR)	92.6	59.5	45.4
**Non-anal SCC**	39	8.7 (6.8–17.8)	46	20.9 (14.0–46.1)	75.2	39.2	25.0
**Head and neck**	12	8.7 (3.1–18.5)	12	23.2 (15.3–49.5)	90.9	40.9	13.6
**Lung**	8	4 (0.8–NR)	8	10.8 (2.2–NR)	41.7	20.8	20.8
**Oesophageal**	4	NR	7	20.9 (9.4–NR)	85.7	34.3	34.3
**Gallbladder**	4	5.8 (2.7–NR)	6	8.9 (1.5–NR)	50.0	33.3	33.3
**Cervix**	4	7.5 (6.8–NR)	6	17.7 (6.1–NR)	80.0	40.0	–

Values in parentheses are 95 per cent confidence intervals. RFS, recurrence-free survival; OS, overall survival; NR, not reached.

**Table 4:** Uni- and multivariable analysis of prognostic factors for recurrence-free and overall survival in liver metastatic anal cancer

	Recurrence-free survival			Overall survival		
	Univariable	Multivariable Cox regression analysis		Univariable	Multivariable Cox regression analysis	
Variable	*P*	Hazard ratio	*P*	*P*	Hazard ratio	*P*
**Patient characteristics**						
Age ≥59 years	0.271			0.926		
Male sex	0.429			0.029	3.00 (0.82–10.89)	0.096
ECOG score ≥2	0.545			0.798		
ASA score ≥3	0.305			0.437		
Charlson co-morbidity index ≥9	0.108	0.43 (0.17–1.09)	0.075	0.483		
**Primary tumour**						
Positive nodal status	0.231			0.105	1.04 (0.32–3.42)	0.938
Tumour stage (T3/T4)	0.253			0.132	2.54 (0.78–8.22)	0.120
**Liver metastases**						
Synchronous	0.843			0.865		
Multiple lesions (>1)	0.400			0.232		
Tumour size (≥32 mm)	0.295			0.428		
Segmental involvement (>1)	0.211			0.033	4.75 (0.85–26.50)	0.076
Systemic chemotherapy	0.477			0.854		
Extrahepatic disease	0.769			**-**		
**Liver resection**						
R1 resection	0.010	6.90 (2.33–20.50)	0.001	0.025	1.62 (0.41–6.37)	0.498

Values in parentheses are 95 per cent confidence intervals. ECOG, Eastern Cooperative Oncology Group.

At the end of follow-up, 51 patients were still alive, resulting in a median OS of 37 (95 per cent c.i. 26 to 50) months, with corresponding 1-, 3-, and 5-year OS of 85, 51 and 36 per cent respectively, for the entire cohort (*Table 3*). A favourable survival was seen for liver-metastatic anal cancer with a median duration of survival and 5-year OS rate of 50 (range 30 to not reached) months and 46 per cent respectively, compared with 21 (range 14–46) months and 25 per cent respectively, in liver-metastatic non-anal cancer, *P* = 0.006 (*Fig. 1b*). The median duration of survival and 5-year OS for men resected for anal cancer liver metastases was 27 months and 25 per cent respectively, and the corresponding survival data for women were 61 months and 53 per cent (*P *=* *0.023). On multivariable analysis on factors influencing survival among anal cancer liver metastases, no individual factor reached significance (*Table 4*). The only factor remaining significantly prognostic of worse OS in the entire cohort was a positive resection margin (hazard ratio 3.21, 95 per cent c.i. 1.24 to 8.30). Excluding the ablated patients from the regression analysis did not alter the result regarding the significant impact of resection margin on RFS (hazard ratio 6.82, 95 per cent c.i. 2.40–19.35) or OS (hazard ratio 3.21, 95 per cent c.i. 1.24 to 8.30). Median duration of survival and OS rate for different primary tumours origin of non-anal SCC are outlined in *Table 3*.

## Discussion

The present study includes the largest cohort to date of patients undergoing liver resection for anal cancer liver metastases and liver metastases from other SCC primaries. This study shows a median OS of 37 months for patients with any SCC liver metastases and a median OS of 50 months in the subgroup of anal cancer liver metastases. Neither extrahepatic metastases, nor synchronous detection of liver metastases, had a negative prognostic impact on OS. A positive resection margin was found to be associated with a more than three-fold increased risk of death in the entire cohort, which emphasizes the importance of achieving a negative resection margin and an important factor to consider when selecting patients for liver resection of SCC liver metastases.

As opposed to the well established practice of liver resection of colorectal and neuroendocrine liver metastases, the benefit of liver resection of SCC metastases and well defined selection criteria for these patients are not fully established^3^^,^^4^^,^^8–13^. Historically, liver resection of SCC has been associated with a poorer outcome with reported median OS following liver resection of 15 to 33 months^1^^,^^2^^,^^9^. Because of the relatively large number of patients with anal cancer liver metastases included in the present study, that group was analysed separately to gain further knowledge of this disease with a known increasing incidence^14^. The liver is the most frequent site of distant metastases in patients with anal SCC, of which approximately 20 per cent develop liver metastases^15^. In the present study, the median OS of 50 months and corresponding 5-year OS of 45 per cent compares favourably to other reports on outcome of patients treated for liver-metastatic anal cancer, reporting a median OS of between 22 and 53 months^2^^,^^5^^,^^14^^,^^16^^,^^17^.

In the present study, R1 resection was the only independent negative prognostic factor after resection, yielding an almost seven-fold increased risk of recurrence. In anal cancer, R1 resection was not an independent prognostic factor for OS. This could potentially be explained by the high proportion of re-resections performed, 11 out of 22 patients with recurrent liver metastases were re-resected. In liver-metastatic anal cancer, the median OS between men and women differed significantly, 27 months compared with 61 months, to the benefit of women. Male sex is associated with a poorer survival for most cancer sites, where anal cancer is one of the cancer sites with the highest male-to-female excess mortality ratio^18^. Unfortunately, the present study lacks information on human papillomavirus prevalence and presence of human immunodeficiency virus, making it difficult to explain the observed differences.

In patients with liver-metastatic colorectal cancer, extrahepatic metastases are no longer considered a contraindication for liver resection^19^. In the present study, a high proportion of patients had extrahepatic metastases at time of liver resection. Extrahepatic disease has been shown to be an independent prognostic factor for reducing both RFS and OS in patients with non-colorectal non-neuroendocrine liver metastases^20^. In the present study, where extrahepatic metastases were treated with potentially curative methods in most patients, no influence on either RFS or OS was found.

Drawing any conclusions on survival outcomes of the cohort of non-anal liver metastases is precarious, but, compared with the limited cases in the existing literature, the small number of each primary tumour site of this study might still contribute important information. Head and neck SCC is the sixth most common cancer worldwide and the liver is the third most common site of metastasis with a reported median survival of about 4 months^21–23^. The median duration of survival of 23 months and 3-year OS rate of 41 per cent respectively, in the 12 patients with head and neck SCC in the present study, compares favourably with previously reported results of 18 months and 24 per cent respectively^1^. Oesophageal cancer still represents an aggressive disease and optimal management of patients with liver oligometastatic oesophageal cancer is still undefined^24^. Adam and colleagues reported a 3-year OS rate of 32 per cent in patients who underwent liver resection for liver metastases of oesophageal cancer (both adenocarcinoma and SCC)^1^. It is to be compared with the 5-year OS rate after liver resection in this study of 34 per cent, potentially indicating a survival advantage in properly selected patients.

Despite being the largest study of its kind, the present study is still limited by the small number of included patients, clearly illustrated in the multivariable analysis with wide confidence intervals. The study is also limited by its retrospective design and lack of comparison group. Unfortunately, the present study lacks information on human papillomavirus prevalence, presence of human immunodeficiency virus and other immunosuppressive disorders. The few patients included per centre obviously introduces a selection bias that cannot be controlled for. The patients in the present series were treated over a period of almost 20 years and certainly chemotherapy regimens, operative technique and diagnostic imaging modalities have changed substantially. In addition, the response to chemotherapy was reported in only 60 per cent of patients receiving preoperative chemotherapy, making it difficult to analyse this factor which could have prognostic importance^25^.

## Funding

J.E. was supported by Region Stockholm (clinical postdoctoral appointment).

*Disclosure*. The authors declare no conflict of interest.
